# Effects and Mechanism of Bufei Yishen Formula in a Rat Chronic Obstructive Pulmonary Disease Model

**DOI:** 10.1155/2014/381976

**Published:** 2014-05-14

**Authors:** Jiansheng Li, Liping Yang, Qin Yao, Ya Li, Yange Tian, Suyun Li, Suli Jiang, Ying Wang, Xinmin Li, Zheng Guo

**Affiliations:** ^1^Henan University of Traditional Chinese Medicine, Zhengzhou 450008, China; ^2^College of Bioinformatics Science and Technology, Harbin Medical University, Harbin 150086, China; ^3^Respiratory Disease Institute, The First Affiliated Hospital of Henan University of TCM, Zhengzhou 450000, China; ^4^Bioinformatics Centre, School of Life Science, University of Electronic Science and Technology of China, Chengdu 610054, China

## Abstract

Bufei Yishen Formula (BYF) has been used for centuries in Asia to effectively treat patients with chronic obstructive pulmonary disease (COPD). This study established a COPD animal model in rats, wherein three groups (control, COPD, and BYF) were used to evaluate the mechanism(s) and curative effect of BYF. Pulmonary function and histomorphology demonstrated that BYF had an evident effect on COPD. Gene microarray was then exploited to analyze the effects of BYF on COPD. ClueGO analysis of differentially expressed genes indicated that BYF improved COPD by regulating expression of interleukins, myosin filament assembly components, and mitochondrial electron transport-related molecules. Moreover, ELISA revealed that expression of several interleukins (*IL1**β**, IL6, IL8, *and* IL10*) was reduced in peripheral blood and bronchoalveolar lavage fluid by BYF treatment. It was concluded that BYF has therapeutic effects on COPD in rats through its effects on interleukin expression and/or secretion. Furthermore, pharmacological or targeted expression of two differentially expressed genes,  * F2R* and*    Sprik1*, might be useful in novel COPD therapies. This study provides the basis for mechanisms of BYF on COPD and new therapeutic drug targets.

## 1. Introduction

Chronic obstructive pulmonary disease (COPD) is a slowly progressive, poorly reversible disease characterized by an abnormal inflammatory response in the lung [1]. The greatest risk factor in COPD development is cigarette smoking [1]. Other risk factors include air pollutants, dust, and inherent susceptibility [1]. The incidence of COPD in general populations is increasing, along with its great burden on public health [2, 3]. Although some glucocorticoids and bronchodilators can alleviate acute onset of COPD, significant side effects exist [4]. For example, *β*2 agonists can induce muscle tremor, tachycardia, sweats, and agitation [5]; theophylline can induce headache, nausea, vomiting, arrhythmias, and seizures [6]; and glucocorticoids can induce osteoporosis [7]. Therefore, the development of new longer lasting, targeted therapeutic strategies is a matter of great urgency.

At present, clinical trials and experimental studies have shown that certain Chinese medicines can effectively treat COPD, by improving pulmonary function, respiratory muscle fatigue, immunity, and lung blood flow [8–10]. Deng [11] found one Chinese herb formula that could improve COPD pathological presentation in a rat model, including inflammatory reactions and airway and pulmonary vasculature plasticity. Moreover, compared to western medicine, many Chinese herbs have few side effects [12]. However, the complexity and various actions of herbal components have limited their application and hindered study of their underlying mechanisms. With the development of high-throughput molecular techniques such as gene microarray, it has become possible to observe the effects of Chinese herbs on genomic expression and seek for their molecular targets.

As a traditional Chinese medicine formula, Bufei Yishen Formula (BYF) has been used for centuries in East Asia to effectively treat patients with COPD. BYF principally employs the use of the herb Huang Qi (*Radix Astragali*) in its herbal formula. Multicentered clinical studies suggest that herbal formulae with Huang Qi were effective for stable COPD [13–18]. However, the quality of these studies has not been evaluated systematically, and no studies have yet explored the molecular mechanism of this particular herbal formula. Our previous studies have shown that BYF has more obvious beneficial effects on clinical symptoms of stable COPD than western medicine. Moreover, apparent positive long-term effects were observed for BYF treatment [19–21]. In this study, a modified COPD rat model was established as described previously by Chen et al. [22] for evaluating the effect(s) of BYF. Three experimental groups (COPD, BYF, and controls) were used to compare pulmonary function and pathology. To investigate the molecular mechanistic effect of BYF on COPD, differentially expressed genes were screened by microarray, and gene function enrichment analyses were conducted.

## 2. Methods

### 2.1. BYF Preparation and Animal Model Establishment

BYF was prepared at the First Affiliated Hospital of Henan University of Traditional Chinese Medicine (Zhengzhou, Henan, China). BYF components were comprised of 15 g Huang Qi (*Radix Astragali*), 15 g Ren Shen (*Radix Ginseng*), 15 g Shanzhuyu (*Fructus Corni*), and 9 g Wuweizi (*Fructus Schisandrae*). All herbs were decocted with water, steam sterilized, and brought to a final concentration of 0.6 g/mL. The BYF was supported by [9] and Chinese patent (number 2011101175781).

Experimental protocols were approved by the Experimental Animal Care and Ethics Committees of the First Affiliated Hospital, Henan University of Traditional Chinese Medicine. Two-month-old Sprague-Dawley rats were purchased from Henan Experimental Animal Center (Henan XK2005-0001) with a body weight of 200 ± 20 g. They were maintained on a 12-hour dark/light cycle with ambient temperature of 25 ± 1°C and relative humidity 50 ± 10%, as well as sufficient food (sterile rat chow) and water (sterile). All rats were anesthetized and sacrificed under the experimental protocols mentioned above and all efforts were made to minimize suffering.

**Figure 1:**
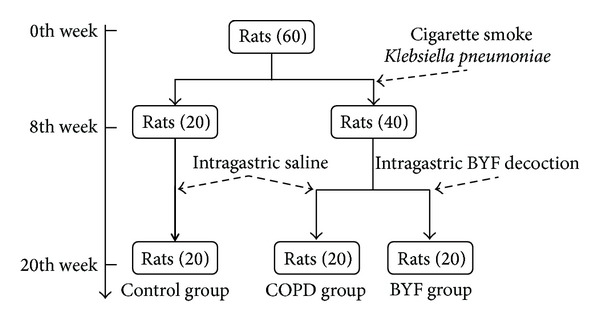
Establishment of COPD animal model.

Sixty Sprague-Dawley rats were randomized and divided into three experimental groups (control, COPD, and BYF) (Figure 1) with equal numbers of males and females in each group. Forty rats underwent intranasal instillation with* Klebsiella pneumoniae* once every 5 days lasting for 8 weeks. Rats were placed in a 300 L smoke box for 30 min with 3 h intervals between smoke treatments, wherein eight cigarettes were burned twice daily in the first two weeks and 15 cigarettes were burned three times daily during weeks 3–8. After 8 weeks of treatment, the 40 COPD-induced rats were randomly divided into two groups: COPD and BYF. The COPD group was administered 2 mL intragastric saline vehicle (0.9%) twice daily during weeks 9–20, and the BYF group was administered 4.44  g/kg/d BYF twice daily during weeks 9–20. The remaining 20 rats were used as a control group with no treatment for the first 8 weeks followed by 2 mL intragastric saline vehicle (0.9%) during weeks 9–20. All rats were weighed weekly to determine dosing and underwent pathologic examination by lung tissue excision (6 samples/group) during the 21st week.

### 2.2. Preparation and Determination of Pulmonary Function and Pathology

Tidal volume (TV), peak expiratory flow (PEF), and 50% tidal volume expiratory flow (EF50) were detected by unrestrained pulmonary function testing plethysmographs (Buxco Inc., Wilmington, NC, USA) conducted every fourth week from weeks 0 to 20. Paraffin-embedded sections of lung tissue were stained with hematoxylin and eosin and images were taken by light microscope (Olympus, Tokyo, Japan).

### 2.3. Interleukin Detection in BALF and Peripheral Blood

Following repeated saline bronchoalveolar lavage (3 mL/lavage), collected BALF underwent 10 min centrifugation (2000 r/min) and the supernatants were used to detect* IL8* and* 10* by ELISA (BOSTER Inc., Wuhan, China). All processes were kept at 4°C. Peripheral blood was collected from rat* aorta abdominalis,* and serum was used to detect* IL1*β*, IL6, IL8*, and* IL10* by ELISA (BOSTER Inc.).

### 2.4. RNA Extraction

Six lung tissue samples were excised from each of the three experimental groups for microarray analysis. Total RNA was isolated from skeletal muscle by Trizol reagent (Invitrogen, Breda, Netherlands) and purified using a Qiagen RNeasy Micro kit (Qiagen, Venlo, Netherlands). RNA quality was verified by Agilent 2100 bioanalyzer (Agilent Technologies, Amsterdam, Netherlands).

### 2.5. Microarray Processing

Purified RNA samples (2 *μ*g ea) were PCR amplified and labeled using an Agilent Quick Amp kit (Agilent Technologies, Santa Clara, CA, USA) and hybridized with Agilent Whole Rat Genome Oligo Microarray (4 × 44 K) in Agilent's SureHyb Hybridization Chambers. After hybridization and washing, processed slides were scanned by an Agilent DNA microarray scanner (part number G2505B) using manufacturer recommended settings.

### 2.6. Data Preprocessing

Raw image data were converted to CEL and pivot files using Agilent Feature Extraction Software version 10.5.1.1. All downstream microarray analyses were performed using Agilent GeneSpring GX software version 11.0. Microarray datasets were normalized in GeneSpring GX using the Agilent FE one-color scenario (mainly median normalization); log 2-transformed data were normalized by quantile normalization and used for comparisons. Differentially expressed genes were identified through fold change (|log ratio| > 1) and Student's *t*-test screening (*P* < 0.05).

ClueGO software [24] was used for gene function enrichment analysis. We incorporated both gene ontology (GO) and the KEGG pathway in ClueGO analysis; GO term fusion and restriction with *P* < 0.05 were chosen, which integrates GO categories and creates a functionally organized GO category network based on overlap between different GO categories and significance. Interleukin expression differences between groups were compared by Student's *t*-test (*P* < 0.01).

## 3. Results

### 3.1. Pulmonary Function and Pathology Improvements in COPD Rats with BYF Treatment

After 20 weeks, control group rats were active and restless, with smooth and burnished fur. Their body mass increased gradually and respiration remained stable. Before BYF treatment, rats in COPD and BYF groups exhibited “spiritual malaise,” which is characterized by appetite suppression and wriggling with gathered fur in the first 8 weeks. The body mass of these rats slowly increased with short respiration accompanied by frequent cough. These symptoms in BYF group rats were significantly alleviated following BYF treatment from weeks 9 to 20.

**Figure 2:**
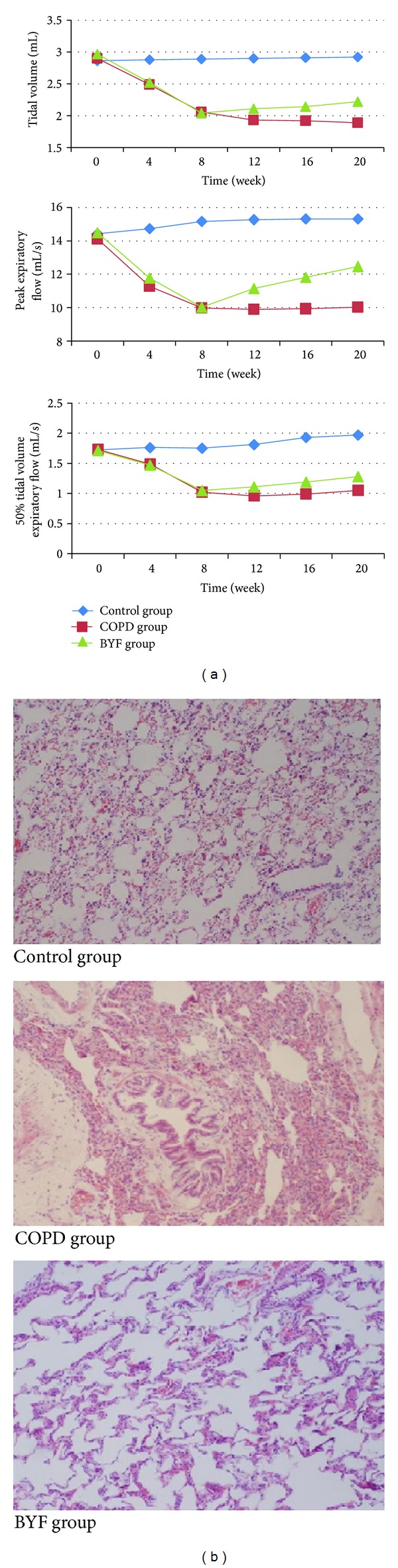
Pulmonary function and pathology in each experimental rat group. (a) TV, PEF, and EF50. (b) Lung histomorphology observations by light microscope. Control group (magnification ×100): pulmonary tissue was smooth and there was no inflammation and cell infiltration, no hyperemia, or no swelling. COPD group (magnification ×100): pulmonary tissue showed upregulation of a severe inflammatory response with visible increases in lymphocytes, monocytes, and neutrophils. Bronchial and pulmonary wall thickness, degree of bronchial stenosis, and alveolar diameter were significantly higher. BYF group (magnification ×100): inflammation around trachea lessened after BYF therapy. The number of inflammatory cells decreased dramatically.

Pulmonary function for all three experimental groups was detected via TV, PEF, and EF50 every four weeks for 20 weeks. TV, PEF, and EF50 were found to be stable in the control group but were dramatically declined in the first eight weeks in the COPD and BYF groups (*P* < 0.05). Following BYF treatment, TV, PEF, and EF50 in the BYF group were significantly improved (all *P* < 0.05; Table 1; Figure 2(a)) compared to rats of COPD group.

Lung histomorphology images from each of the three experimental groups are shown in Figure 2(b). Compared to controls, COPD rats showed upregulation of a severe inflammatory response with visible increases in lymphocytes, monocytes, and neutrophils. Bronchial and pulmonary wall thickness, degree of bronchial stenosis, and alveolar diameter were significantly higher in the COPD group, while the alveolar count per unit area was significantly lower compared to control rats. These COPD-related phenomena were dramatically relieved with BYF treatment. Furthermore, BYF treatment significantly alleviated the inflammatory response, as shown by a significant decrease in the number of inflammatory cells present in lung tissues.

### 3.2. Gene Microarray Data Analysis

**Figure 3:**
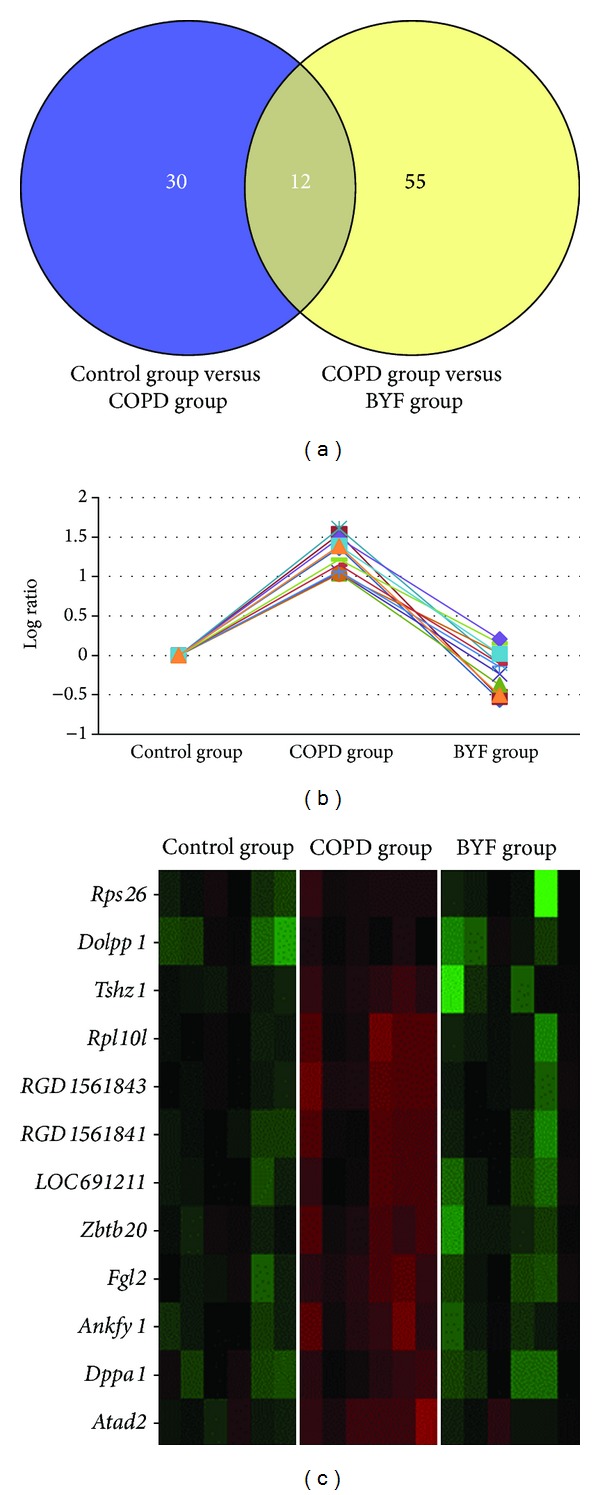
Venny plot of DEGs between each experimental group and expression of 12 overlapping genes. (a) Venny plot of two DEG lists (COPD versus controls and COPD versus BYF). (b) Expression of 12 overlapping genes from all three rat groups. (b) Gene expression heat map of 12 overlapping genes.

To investigate the molecular mechanism of BYF on COPD pathogenesis, we randomly chose eighteen samples from the three experimental groups (six samples from each experimental group) for gene expression experiments. Using |log ratio|> 1 and *P* < 0.05, 42, and 67, differentially expressed genes (DEGs) were detected between control and COPD groups as well as COPD and BYF groups (Figure 3(a); see Table S1 in Supplementary Material available online at http://dx.doi.org/10.1155/2014/381976), respectively. Twelve genes were overlapped between the two DEG lists (control versus COPD and COPD versus BYF). As shown in Figures 3(b) and 3(c), all 12 overlapped genes were increased in COPD rats and then decreased in the BYF group. GO enrichment analysis by Bingo software [23] showed that these 12 overlapping genes were involved in ribosomal structure/function, gene expression, translation, and negative regulation of RNA splicing (Table 2). In particular, four overlapped genes (*RPL10L, RPS26, RGD1561843, *and* RGD1561841*) are located in the ribosome. This indicates that BYF treatment may reduce mRNA translation and translational editing in COPD.

### 3.3. ClueGO Analysis of Differentially Expressed Genes

**Figure 4:**
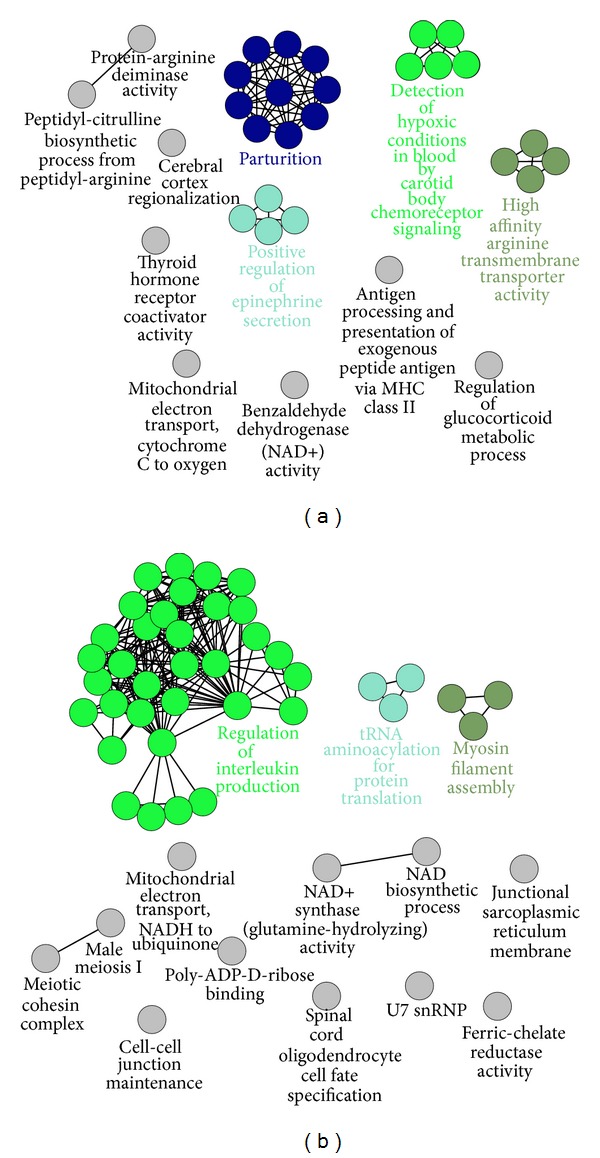
ClueGO analysis for DEGs. (a) ClueGO analysis for DEGs between COPD and control groups. (b) ClueGO analysis for DEGs between COPD and BYF groups.

To facilitate understanding of the biological implications of DEGs, functional enrichments were performed by ClueGO [24], which incorporates gene ontology and KEGG pathway annotation. ClueGO integrates GO categories and creates functionally organized GO category networks based on overlap between different GO categories and statistical significance. In line with previous studies of COPD and anoxic and oxidative stress [25], DEGs detected between our COPD and control groups were largely implicated in detection of hypoxic conditions in blood by carotid body chemoreceptor signaling, mitochondrial electron transport, cytochrome C to oxygen, and benzaldehyde dehydrogenase (NAD+) activity (Figure 4(a)). An especially interesting finding of this analysis was the significant enrichment for terms involved in parturition.

Significant GO and DEG pathway terms between COPD and BYF groups are presented in Figure 4(b). These DEGs were mainly enriched in terms involved in regulation of interleukin production, mitochondrial electron transport, NADH to ubiquinone, NAD+ synthase activity, NAD biosynthesis, poly-ADP-D-ribose binding, and myosin filament assembly. Because COPD is known to be accompanied by pulmonary inflammation, oxidative stress, and muscle fiber dysfunction [25–27], BYF treatment may improve these COPD symptoms by regulating the above GO term functions.

**Figure 5:**
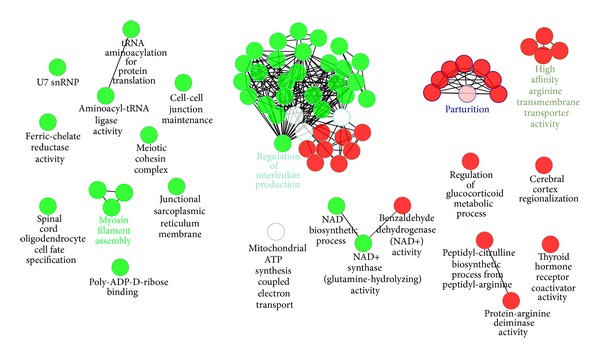
Comparative ClueGO analysis for two DEG lists (COPD versus controls and COPD versus BYF). Red node: functions only enriched by DEGs between control and COPD groups; green node: functions only enriched by DEGs between COPD and BYF groups; white node: functions enriched by both DEG lists.

Comparative ClueGO analysis between the two DEG lists (COPD versus control and COPD versus BYF) demonstrated that both DEG lists shared at least three relatively enriched GO terms in common including interleukin production, NAD/NADH, and mitochondrial electron transport-related function (Figure 5).

### 3.4. Interleukin Expression Level in Serum and BALF

**Figure 6:**
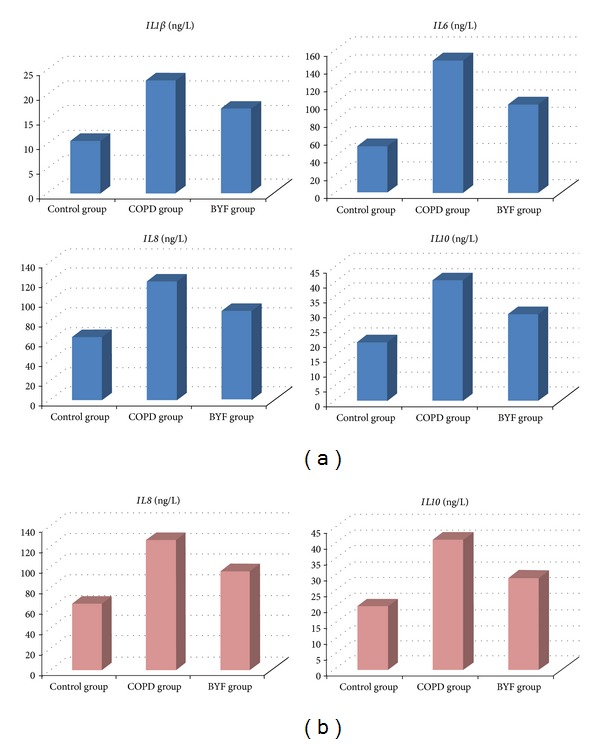
Interleukin expression levels in BALF and serum. (a) Expression level of* IL1*β*, IL6, IL8,* and* IL10* in serum. (b) Expression level of* IL8* and* IL10* in BALF.

Interleukin production and inflammatory functions were evaluated by ClueGO analysis [24], which demonstrated increases in the levels of several common serum (*IL1*β*, IL6, IL8,* and* IL10*) and BALF (*IL8* and* IL10*) interleukins by ELISA. At the end of 20 weeks, serum levels of* IL1*β*, IL6, IL8, *and* IL10* in the COPD group were significantly higher than in the control group (*P* < 0.01), while those in the BYF group were significantly lower than the COPD group (*P* < 0.01). Similar to serum,* IL8* and* IL10* levels were also decreased with BYF treatment in BALF (*P* < 0.01; Table 3; Figure 6(b)). These findings indicate that BYF can alleviate COPD inflammation by reducing interleukin levels.

## 4. Discussion

Although technological advances have been made, there are still considerable complications associated with human tissue sampling. Animal models that imitate COPD pathogenesis avoid the risks of human experimentation [28–30] and provide a basis for evaluating the effectiveness of new therapeutic strategies. At present, COPD animal models that are consistent with human COPD are available [9, 10]. In this study, rat COPD was induced by cigarette smoking in combination with repeated instillation of* Klebsiella pneumonia*. COPD lung tissue pathology by light microscope demonstrated that lesions in the rat model closely resemble those occurring in human COPD.

The complexity and varying action of components of Chinese herbs have limited their extensive utilization and hindered study of their underlying molecular mechanism(s). With development of high-throughput molecular techniques such as gene microarray, it is possible to examine the effects of Chinese herbs on genomic expression. To evaluate the effect of BYF on COPD in the present study, we established three groups of rats (COPD, BYF, and controls) and compared their pulmonary function and pathology. Results showed that BYF treatment can significantly improve pulmonary function and lung tissue pathology of COPD rats. Gene expression profiles were used to explore the multitarget characteristics of BYF treatment. Gene function enrichment analysis indicated that the BYF can improve COPD through mitochondrial electron transport-related molecules (NAD/NADH/ADP), regulation of interleukin expression, and myosin filament assembly components, relating to muscle dysfunction. Many studies suggested that COPD was related to respiratory, diaphragmatic, and skeletal muscle dysfunction [27, 31–33]. Mitochondrial electron transport-related molecules (NAD/NADH/ADP) are also in relation with redox reactions and oxidative stress, which are known to be the key factors in COPD [33, 34]. In addition, regulation of interleukin expression, involved in inflammation, is also a symptom of COPD [35–37].

Chronic airway inflammation is a key aspect in the pathogenesis of COPD, associated with almost all structural and functional damage of airway and lung tissue [38]. Previous studies showed that members of inflammatory cells such as neutrophils, lymphocytes, and alveolar macrophages were aggregated in blood, sputum, BALF, and bronchial mucosa in stable COPD patients [39]. Inflammatory cells also release various cytokines, including* IL1*β*, IL6, IL8,* and* IL10,* which play an important role in inflammatory response. In this study, we detected expression of* IL1*β*, IL6, IL8,* and* IL10* in peripheral blood and BALF. Results show that all four interleukins were reduced by BYF treatment in COPD rats, which was consistent with the gene function enrichment analysis.

**Figure 7:**
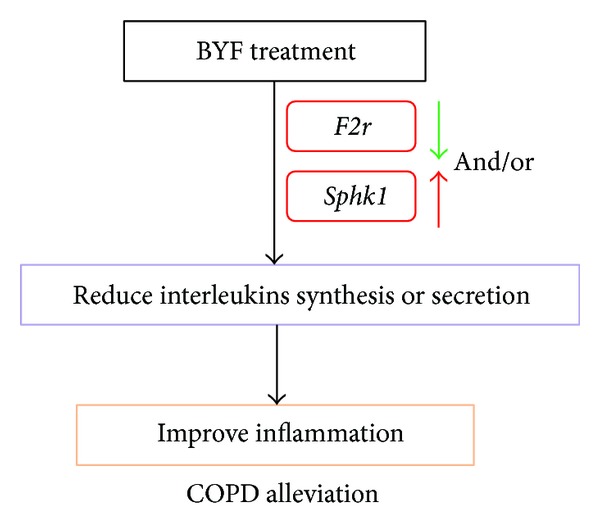
Mechanism of BYF treatment for COPD.

In gene expression profiles, no difference was found for interleukin expression among treatment groups. However, expression changes of several interleukins were detected in BALF and serum, suggesting that BYF treatments cannot downregulate interleukin transcription but reduce translation and/or protein secretion. Gene function enrichment analysis of 12 overlapping DEGs (between control versus COPD and COPD versus BYF groups) indicates that BYF treatment can reduce translation and RNA splicing in COPD. As confirmation, we focused on two DEGs (*F2R* and* Sphk1*) that are related to the regulation of interleukin production. Interestingly, studies by Gigante et al. [40, 41] showed that* F2R* haplotypes influence serum* IL6* levels in humans and regulate* IL6* synthesis and production in endothelial cells. In other words,* F2R* haplotypes may influence* IL6* synthesis and secretion. Besides, the human protein-protein interactions predictions (PIPs) database predicts that* F2R* may physically interact with* IL8* [42], while Li et al. [43] found that* Sphk1* expression and activity could reduce* IL1*β** and* IL6* concentrations in the serum. Therefore, we speculate that* F2R* and* Sphk1* can regulate the synthesis and/or secretion of certain interleukins in our COPD rat models, while BYF treatment does not influence transcription but reduces interleukin translation or secretion by regulating expression of* F2R* and* Sphk1*, thereby improving COPD-related inflammation (Figure 7).

## Supplementary Material

Table S1: DEGs between experimental groups. (A) DEGs between control and COPD groups. (B) DEGs between COPD and BYF groups.
Computational method of the DEGs: after microarray experiments, raw image data were converted to CEL and pivot files using Agilent Feature Extraction Software version 10.5.1.1. All downstream microarray analyses were performed using Agilent GeneSpring GX software version 11.0. Microarray datasets were normalized in GeneSpring GX using the Agilent FE one-color scenario (mainly median normalization); Log 2-transformed data were normalized by quantile normalization and used for comparisons. Differentially expressed genes were identified through fold change (|log ratio|>1) and Student's t-test screening (P < 0.05).

## Figures and Tables

**Table 1:** TV, PEF, and EF50 in three experimental rat groups ($\bar{x}\pm S$).

Index	Group	0 weeks	4 weeks	8 weeks	12 weeks	16 weeks	20 weeks
TV (mL)	Control	2.86 ± 0.46	2.88 ± 0.33	2.89 ± 0.34	2.90 ± 0.32	2.91 ± 0.36	2.92 ± 0.24
	COPD	2.90 ± 0.38	2.49 ± 0.24	2.06 ± 0.22	1.93 ± 0.22	1.92 ± 0.30	1.89 ± 0.36
	BYF	2.97 ± 0.46	2.52 ± 0.21	2.04 ± 0.33	2.11 ± 0.18	2.14 ± 0.25	2.22 ± 0.36

PEF (mL/s)	Control	14.43 ± 2.69	14.72 ± 2.48	15.16 ± 1.99	15.25 ± 2.02	15.31 ± 1.75	15.30 ± 2.24
	COPD	14.11 ± 2.47	11.28 ± 2.00	9.99 ± 2.33	9.90 ± 1.96	9.93 ± 2.40	10.02 ± 2.18
	BYF	14.49 ± 2.53	11.78 ± 1.96	10.03 ± 2.48	11.15 ± 2.51	11.81 ± 2.61	12.35 ± 2.84

EF50 (mL/s)	Control	1.72 ± 0.16	1.76 ± 0.12	1.75 ± 0.15	1.81 ± 0.17	1.93 ± 0.28	1.97 ± 0.21
	COPD	1.73 ± 0.11	1.49 ± 0.13	1.02 ± 0.17	0.96 ± 0.20	0.99 ± 0.17	1.05 ± 0.17
	BYF	1.71 ± 0.12	1.47 ± 0.20	1.05 ± 0.20	1.11 ± 0.22	1.17 ± 0.28	1.24 ± 0.28

**Table 2:** Bingo analysis of 12 overlapping genes [23].

GO term	*P* value	FDR	Gene
Structural constituent of ribosome	1.31*E* − 04	1.16*E* − 02	*RPL10L/RPS26/RGD1561843/RGD1561841 *
Ribosome	1.76*E* − 04	1.16*E* − 02	*RPL10L/RPS26/RGD1561843/RGD1561841 *
Translation	2.65*E* − 04	1.17*E* − 02	*RPL10L/RPS26/RGD1561843/RGD1561841 *
Ribonucleoprotein complex	5.64*E* − 04	1.86*E* − 02	*RPL10L/RPS26/RGD1561843/RGD1561841 *
Structural molecule activity	7.05*E* − 04	1.86*E* − 02	*RPL10L/RPS26/RGD1561843/RGD1561841 *
Soft palate development	2.16*E* − 03	3.55*E* − 02	*TSHZ1 *
Negative regulation of RNA splicing	2.16*E* − 03	3.55*E* − 02	*RPS26 *
Macromolecular biosynthesis	2.42*E* − 03	3.55*E* − 02	*RPL10L/RPS26/RGD1561843/RGD1561841 *
Gene expression	3.36*E* − 03	4.44*E* − 02	*RPL10L/RPS26/RGD1561843/RGD1561841 *

**Table 3:** Interleukin expression levels in serum and BALF in the 20th week.

Interleukin	Group	Serum (ng/L)	BALF (ng/L)
*IL1 β*	Control	10.62 ± 1.68	
	COPD	22.81 ± 1.34	
	BYF	17.13 ± 0.71	

*IL6 *	Control	52.48 ± 3.42	
	COPD	148.71 ± 4.72	
	BYF	99.32 ± 4.45	

*IL8 *	Control	64.06 ± 8.17	64.60 ± 8.77
	COPD	120.33 ± 7.15	126.60 ± 6.89
	BYF	90.33 ± 4.37	96.52 ± 6.90

*IL10 *	Control	19.72 ± 2.93	20.18 ± 2.43
	COPD	40.50 ± 2.13	41.16 ± 2.58
	BYF	29.18 ± 1.55	29.12 ± 1.54
